# Correlation Analysis of Refractive and Visual Quality after Wavefront-Optimized Laser In Situ Keratomileusis for 50% and 100% Angle Kappa Compensation

**DOI:** 10.1155/2020/9873504

**Published:** 2020-10-05

**Authors:** Xin-Yu Ru, Zheng-Ri Li, Cheng-Lin Li, Hong Cui, Wen-Qing Deng, Shu-Hua Lin, Yu-Jie Jia, Ying-Jun Li

**Affiliations:** Department of Ophthalmology, Affiliated Hospital of Yanbian University, Yanji 133000, Jilin Province, China

## Abstract

**Purpose:**

To analyze the distribution of the offset between the pupil center and the coaxially sighted corneal light reflex (*P-Dist*), the effects of 50% and 100% angle kappa adjustments on refractive and visual quality in patients with moderate myopia were investigated.

**Methods:**

A randomly selected 254 patients (254 eyes) with moderate myopia who underwent femtosecond laser-combined LASIK were examined. During the operation, the *P-Dist* of the patients was recorded by the *x-* and y-axis eyeball-tracking adjustment program of the WaveLight Eagle Vision EX500 excimer laser system. Preoperatively and 3 months postoperatively, the WaveLight® ALLEGRO Topolyzer was used to measure the pupil size and center position, and the wavefront sensor was used to measure the wavefront aberrations. The visual function tester (OPTEC 6500) measured contrast sensitivity.

**Results:**

The average *P-Dist* was 0.220 ± 0.102 mm. When the *P-Dist* >0.220 mm, the postoperative residual cylinder was 0.29 ± 0.34 D in the group with the 50% adjustment and 0.40 ± 0.32 D in the 100% group, which was significantly higher than the 50% group (*P*=0.036). The coma was 0.21 ± 0.17 *μ*m in the 50% adjusted group and 0.34 ± 0.25 *μ*m in the 100% group, which was significantly higher than that in the 50% group (*P*=0.021). At the 1.5 c/d spatial frequency, contrast sensitivity in the adjusted 100% group was significantly lower than that in the 50% group under visual glare conditions (*P*=0.039).

**Conclusion:**

The postoperative visual acuity and spherical equivalent were not affected in the two groups. However, when *P-Dis*t >0.220 mm, the residual astigmatism and coma were lower in the 50% group. Individualized operations for those with moderate myopia and large-angle kappa in which 100% adjustment is chosen may not result in a better visual quality effect than 50%.

## 1. Introduction

The human eye is a complex optical system with multiple axes (visual axis, optical axis, pupillary axis, etc.) and multiple angles (angle kappa, angle alpha, etc.) [1]. The angle kappa is defined as the angle between the visual axis and pupillary axis. In the individualization of corneal refractive surgery, the ideal excimer laser cutting center should completely overlap with the visual axis because the visual axis is difficult to determine during the operation. The eye-tracking system usually locates and tracks the pupil (pupil center), but the pupil center is different from the visual axis [1]. If angle kappa adjustment is not considered during the pupil positioning and tracking scan, it will lead to surgically induced decentration [2], resulting in an increase in higher-order aberrations (HOAs) after surgery [3]. Therefore, adjusting the excimer laser cutting center from the pupil center to the visual axis to compensate for the offset effect of angle kappa has become the consensus among corneal refractive surgeons [4, 5].

The current individualized laser cutting technology for angle kappa adjustments corrects only the offset between the static pupillary axis and visual axis but does not take factors such as dynamic changes in the pupil, cornea, and lens into account. However, angle kappa is not a fixed value, and it will change under different conditions. It can be affected by factors such as light during surgery, surgical stimulation, emotional tension, and convergence adjustment caused by watching the indicator at close range [6–8]. According to the data regarding changes in angle kappa, can we obtain individual kappa angle adjustment vector percentages, find the cutting center point closest to the visual axis, and ensure that each excimer laser spot is in the exact position?

In this study, by analyzing the distribution rule of the vector between the pupil center and the coaxially sighted corneal light reflex, it is intended to analyze the effect of individualized excimer laser in situ keratomileusis with different angle kappa compensation, through the percentages of 50% and 100% angle kappa compensation, on diopter and visual quality in eyes with moderate myopia. The study provides a reliable theoretical and experimental basis for the design of an optimized femtosecond laser combined with excimer laser in situ keratomileusis that meets the optical characteristics of individual human eyes.

## 2. Materials and Methods

### 2.1. Patient Selection

Two hundred and fifty four patients (254 eyes; the right eyes) with moderate myopia who underwent femtosecond laser-assisted in situ keratomileusis (FS-LASIK) at the Department of Ophthalmology in Affiliated Hospital of YanBian University from January to May 2019 were randomly selected for preoperative and postoperative follow-ups at 3 months. The range of preoperative spheres was −3.00 to −6.00 D. The cylinder was 0 to 1.50 D, and the refractive state was stable in the last 2 years (the annual change was less than 0.5 D) (Table 1). Before the operation, the subjects were randomly assigned to 50% (127 patients, 127 eyes) and 100% (127 patients, 127 eyes) angle kappa adjustment groups. The use of soft contact lens was stopped for more than 2 weeks, and the use of rigid permeable contact lens was stopped for more than 1 month. The corneal thickness was ≥480 *μ*m before surgery and postoperative residual stromal bed thickness was >300 *μ*m. The exclusion criteria included subjects with ocular pathology, ophthalmic disorders, amblyopia, strabismus, previous intraocular surgery, laser treatment, or retinal complications. Informed consent was obtained from all subjects using a consent form approved by the Institutional Review Board of the Affiliated Hospital of Yanbian University.

### 2.2. Surgical Techniques

All patients underwent slit-lamp examination. Computer optometry and retinoscopy were used for objective optometry and cycloplegic refraction, comprehensive optometry, intraocular pressure (IOP), corneal thickness, eye axis, and fundus examination. At the preoperative and postoperative l-week, 1-month, and 3-month examinations, a WaveLight® ALLEGRO Topolyzer (WaveLight Laser Technologies, AG, Erlangen, Germany) was used to measure the pupil size and center position, and a wavefront sensor (VISX WaveScan) was used to measure HOAs of the eyeball (including 3–6 total higher-order aberrations, spherical aberrations, coma, and trefoil) under the condition of a normal pupil diameter of 5 mm in the darkroom. The Optec 6500 Vision Tester (Stereo Optical Co., Chicago, IL, USA) was used to measure the contrast sensitivity at 5 spatial frequencies (1.5, 3.0, 6.0, 12.0, and 18.0 c/d). The preoperative examination, operation, and postoperative observations were made by the same physician.

### 2.3. Evaluation Index

The WaveLight FS200 femtosecond laser (Alcon Laboratories, Inc., Fort Worth, TX) was used to produce 110 *μ*m corneal flaps with a diameter of 8.5 mm. The WaveLight EX500 excimer laser (Alcon Laboratories, Inc.) was used for excimer laser cutting with a wavefront aberration optimized cutting program. The same surgeon performed all surgeries, and the targeted refraction was +0.50 diopter (D).

The following procedures were used. The eye was conventionally disinfected, and the eyelid was opened with a blepharostat. After making a flap with the femtosecond laser, the patients were asked to lie flat and to watch the upper green indicator light. The examiner could see the reflective point of the corneal vertex (coaxial corneal reflection point) and the red reflection in the center of the pupil (optical axis center; origin of Cartesian coordinate system) under the microscope, while adjusting the illumination of the operating microscope and indoor lighting to keep the pupil size consistent with the preoperative examination; if the actual pupil in the treatment image differed in diameter by more than 20% from the diagnostic image, it was possible to modify the actual pupil size and diameter by changing the lighting conditions using the “microscope/op field illumination brightness knob.” The *x*- and *y*-axis eye-tracking adjustment program of the EX500 excimer laser system was used to record the *P-Dist* (the offset between the pupil center and the coaxially sighted corneal light reflex) while the patient was supine. The 50% and 100% *P-Dist* adjustment was manually entered into the excimer laser device. The excimer laser cutting center was moved from the pupil center to the direction of the visual axis (coaxially sighted corneal light reflex).

The diameter of the optical cutting was 6.5 mm, and laser cutting was performed according to a predesigned procedure. After completion, the flap was reset, the residue under the flap was washed, and the eyelid opener was removed.

### 2.4. Statistical Methods

All statistical analyses were performed using SPSS 21 for Windows (SPSS Inc., Chicago, IL, USA). Independent sample *t*-tests were used to compare the *P-Dist* indexes in the 50% and 100% groups. Paired *t*-tests were used to compare the preoperative and postoperative values, and a *P* value of <0.05 was considered to indicate a significant difference.

## 3. Results

### 3.1. The Distribution of Decentration between the Pupil Center and the Coaxially Sighted Corneal Light Reflex

**Figure 1:**
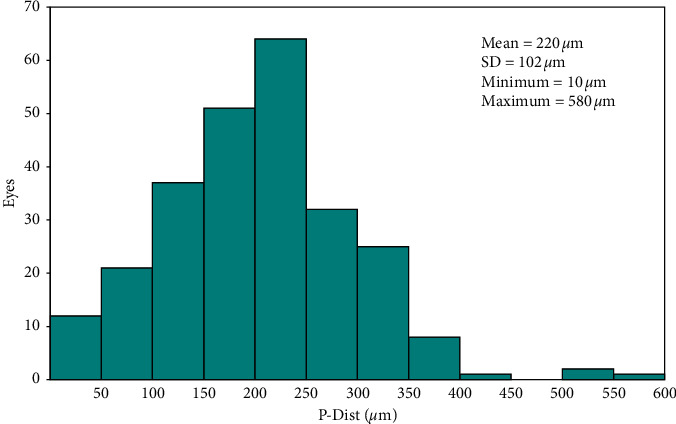
Decentration of the *P-Dist* (distance between the pupil center and the coaxially sighted corneal light reflex).

**Figure 2:**
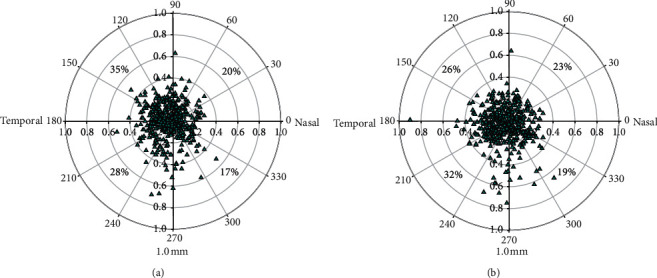
Decentration of the pupil center under (a) photopic and (b) mesopic conditions (the center of the coordinate is the geometric center of the cornea, and the cyan points are pupil centers).

The distribution of decentration between the pupil center and the coaxially sighted corneal light reflex was 0.220 ± 0.102 mm (range: 0.010 to 0.580 mm) with 32% of eyes ≤0.15 mm, 88% of eyes ≤0.30 mm, and 98% of eyes ≤0.45 mm (Figure 1); there were 130 eyes (50%: 62 eyes, 100%: 68 eyes) in the *P-Dist* < 0.220 mm (small-angle kappa) and 124 eyes (50%: 65 eyes, 100%: 59 eyes) in the *P-Dis*t > 0.220 mm (large-angle kappa), with the 50% group at 0.215 ± 0.125 mm and the 100% group at 0.226 ± 0.97 mm. There was no significant difference between the two groups (*P*=0.641). The distribution of decentration between the corneal center and the pupil center under photopic and scotopic conditions showed that under photopic conditions, the superior temporal region accounted for 35%, the inferior temporal region accounted for 28%, the superior nasal region accounted for 20%, and the inferior nasal region accounted for 17%; under scotopic conditions, the superior temporal region accounted for 26%, the inferior temporal region accounted for 32%, the superior nasal region accounted for 23%, and the inferior nasal region accounted for 19% (eye ratio) (Figure 2).

### 3.2. Comparison of Postoperative Visual Acuity and Diopter

There was no significant difference between the 50% (0.02 ± 0.01) and 100% (0.03 ± 0.02) groups in postoperative distance-corrected visual acuity (logMAR acuity) (*t* = 0.009, *P*=0.954). Postoperatively, the two groups showed slight hyperopia drift that accounted for more than 91% within ± 0.50 D. When the *P-Dist* < 0.220 mm, the residual postoperative cylinder was 0.31 ± 0.28 D in the 50% group and 0.34 ± 0.41 D in the 100% group. There was no significant difference between the two groups (*t* = −0.339, *P*=0.412). When P*-Dist* > 0.220 mm, the residual postoperative cylinder was 0.29 ± 0.34 D in the 50% group and 0.40 ± 0.32 D in the 100% group, which was significantly higher than that in the 50% group (*t* = −2.047, *P*=0.036) (Table 2).

More eyes achieved zero astigmatism in the 50% group (53 eyes, 41.7%) than in the 100% group (40 eyes, 31.5%). More eyes had astigmatism greater than 0.75 D in the 100% group (9 eyes, 7.1%) than in the 50% group (2 eyes, 1.6%). There was a significant difference in the distribution of the postoperative cylinder between the 50% and 100% groups (*χ*^2^ = 5.64, *P*=0.042).

### 3.3. Higher-Order Aberration Analysis

There were no significant differences in preoperative HOA, RAS, spherical aberrations, coma, and trefoil at different *P-Dist* values between the 50% and 100% groups (*P* > 0.05) (Table 3). When the *P-Dist* < 0.220 mm, the coma in the 50% group was 0.17 ± 0.12 *µ*m, and the coma in the 100% group was 0.22 ± 0.19 *µ*m. There were no significant differences between the two groups (*t* = −1.424, *P*=0.256). However, when *P-Dist* > 0.220 mm, the coma was 0.21 ± 0.17 *µ*m in the 50% group and 0.34 ± 0.25 *µ*m in the 100% group, which was significantly greater than that in the 50% group (*t* = −2.322, *P*=0.021); in the 100% group, the coma was 0.22 ± 0.19 *µ*m in those with *P-Dist* < 0.220 mm and was 0.34 ± 0.25 *µ*m in those with *P-Dist* > 0.220 mm, which was significantly different (*t* = −2.017, *P*=0.045).

### 3.4. Contrast Sensitivity Comparison

**Figure 3:**
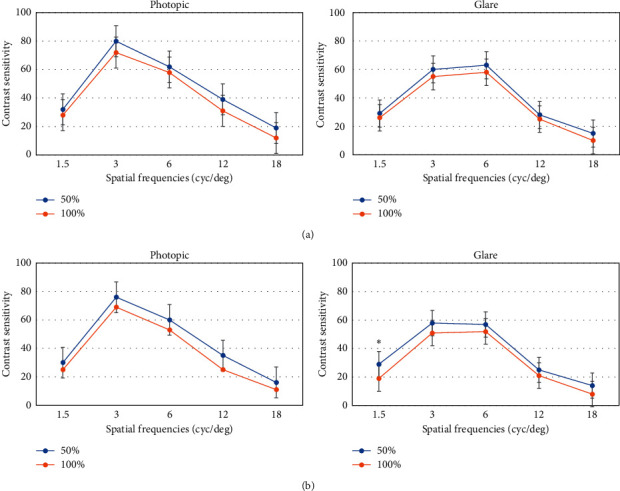
Comparison of postoperative contrast sensitivity between groups under photopic and glare conditions ((a) *P-Dist* < 0.220 mm; (b) *P-Dist* > 0.220 mm). ^*∗*^*P* < 0.05.

When the *P-Dist* < 0.220 mm, there was no significant difference in the contrast sensitivity between the 50% and 100% groups under conditions of photopic vision and photopic glare when adjusted across 5 spatial frequencies (*P* > 0.05). However, when the *P-Dist* > 0.220 mm, contrast sensitivity in the 100% group was significantly lower than that in the 50% group under the condition of a 1.5 c/d spatial frequency and postoperative photopic glare (*t* = 3.673, *P*=0.039) (Figure 3).

## 4. Discussion

Although compensation for angle kappa combined with various modes of personalized LASIK has a good theoretical basis, there is still a significant gap between the actual and ideal visual quality [9, 10]. The currently available [11] angle kappa adjustment is compensated according to the vector percentage between the pupil center and the corneal coaxial reflection point, but the input value is a fixed decentration.

Pande and Hillman [12] showed that the corneal coaxial reflective point was the ideal excimer laser cutting center because the corneal coaxial reflective point is the closest point to the visual axis and is not affected by changes in the pupil size and center position, with an average of 0.02 mm. Therefore, the angle kappa can be understood as the distance between the pupil center and the corneal coaxial reflective point. The corneal reflection point will be more accurate and stable [13] if the errors in the patient's eyeball swing and excimer laser tracking system can be supplemented with limbal vascular network tracking during the operation. In this study, we measured the distance between the pupil center and the corneal coaxial reflection point, showing that the average *P-Dist* was 0.220 mm, the minimum was 0.010 mm, and the maximum was 0.580 mm. Accordingly, based on the above average value, we divided individuals into *P-Dist* groups with >0.220 mm (large-angle kappa) and <0.220 mm (small-angle kappa), and we tried to verify which compensation proportion, either 50% or 100% ablation centration, was closer to the visual axis based on an exploration of the effects of personalized excimer laser in situ keratomileusis with different angle kappa compensation levels on the diopter and visual quality in those with moderate myopia.

This study showed that there was no significant difference in postoperative uncorrected visual acuity and spherical equivalent between the 50% and 100% groups. The residual diopters in the 50% and 100% groups were very small, and the equivalent spherical mirrors presented slight hyperopia drift that accounted for more than 91% of those within +0.50 D. The 50% and 100% groups had overcorrection after cutting by a WaveLight EX500 excimer laser, which is consistent with the results of an evaluation of that excimer laser in treating myopia [10]. When the *P-Dist* > 0.220 mm, the postoperative residual cylinder power was 0.29 ± 0.34 D in the 50% group and 0.40 ± 0.32 D in the 100% group. The 50% group had less residual astigmatism than the 100% group. In addition, we found, regarding the astigmatism data, that more eyes achieved zero residual astigmatism, but fewer eyes had astigmatism greater than 0.75 D in the 50% group than in the 100% group.

When *P-Dist* > 0.220 mm, the coma in the 100% group was significantly higher than that in the 50% group, and contrast sensitivity in postoperative visual glare conditions at low spatial frequency was also lower than that in the 50% group. The increase in spherical aberration and trefoil was attributed to the ablation profile; however, the induced coma could have been caused by the position of the ablation centration points [14] and astigmatism [4]. Mrochen et al. [15] also reported that subclinical decentered ablation (<1.0 mm) was the main reason for the increase in postoperative coma. We speculate that although the adjustment in the 100% group did not affect the postoperative visual acuity, when the large kappa angle (*P-Dist* > 0.220 mm) was adjusted to 100%, the offset between the cutting center and the visual axis was larger than that in the 50% group, which may be because the laser cutting center exceeded the visual axis center [16]; the increase in the incident oblique beam led to an increase in astigmatism and an increase in postoperative coma, which led to a decrease in visual quality, such as glare, and a decrease in contrast sensitivity.

In this study, eye-tracking technology based on image processing with the noninterference pupillary-corneal reflex method was used to track the pupil center of the operative eye, and the direction of the visual axis could be estimated by calculating the vector between the pupil center and the coaxially sighted corneal light reflex [17]. With the locked pupil center as the reference of the Cartesian coordinate system, the adjusted vector ratio of 50% and 100% was a fixed value, rather than a value that changed with the dynamic pupil. This study also showed that the size and position of the pupil center under photopic and scotopic conditions were dynamic changes. For the center position of the pupil, the superior temporal region accounted for 35% under photopic conditions, and the inferior temporal region accounted for 32% under scotopic conditions. The ideal decentration should refer to the pupil size and center position and the dynamic changes in angle kappa to obtain the individual curve-shifted pupil centers [18]. During the operation, the patient's pupil dynamics were monitored, and the angle kappa was adjusted to calibrate the cutting center in real time.

The larger the angle kappa is, the greater the distance between the pupil center and the coaxially sighted corneal light reflex will be. Theoretically, when 100% angle kappa is compensated, the ablation centration is closer to the visual axis, but we found that there was still a gap between the actual and expected visual quality. It is speculated that factors such as dynamic changes in the pupil center position caused by lighting, emotional tension, surgical stimulation, and adjustment of radial convergence during the operation were involved, which further confirm the importance of the accurate positioning of the ablation centration point [19] and the necessity of reasonable compensating for the percentage under different angle kappa states [20].

This study is the first to investigate the distribution of decentration between different pupil centers and the coaxially sighted corneal light reflex. The refraction, HOAs, and contrast sensitivity results were compared by adjusting the vector ratio of 50% and 100% *P-Dist*. It was shown that neither group had central vision affected, and there was no difference in equivalent spherical lens. However, in the 50% group, there was less residual astigmatism and coma. Therefore, both 50% and 100% *P-Dist* adjustments were effective in achieving good postoperative visual acuity. However, in moderate myopia patients with large-angle kappa, choosing 100% adjustment may not result in better visual quality than 50% adjustment. In the individualized operation of moderate myopia with a large kappa angle, choosing 100% adjustment may not result in a better visual quality effect than 50% adjustment. In addition, the percentage comparison with other angle kappa results, the correspondence between angle kappa compensation and wavefront optimization, and the correspondence with visual quality need to be further explored.

## Figures and Tables

**Table 1:** Patient demographics and characteristics.

	50% group	100% group	*P* value
Age (years)	23.51 ± 6.82	24.68 ± 4.51	0.954
Spherical equivalent (D)	−5.05 ± 1.35	−5.21 ± 1.56	0.292
Sphere (D)	−4.62 ± 1.05	−4.85 ± 1.26	0.685
Cylinder (D)	−0.87 ± 0.61	−0.72 ± 0.59	0.198
Corneal *K*-value (D)	42.61 ± 1.23	43.56 ± 1.54	0.294
Corneal thickness (*μ*m)	529.72 ± 32.26	535.24 ± 41.23	0.586
Intraocular pressure (mmHg)	13.42 ± 1.37	12.08 ± 2.04	0.319
Pupil diameter (mm)			
Photopic	3.25 ± 0.49	3.52 ± 0.37	0.216
Mesopic	6.24 ± 0.71	6.38 ± 0.56	0.659
Axial length (mm)	26.71 ± 1.59	26.95 ± 1.82	0.482

**Table 2:** Comparison of diopters between different *P-Dist* groups at 3 months.

Category	*P-Dist* < 0.220 mm	*P-Dist* > 0.220 mm	*t* value	*P* value
Spherical (D)				
50% group	0.24 ± 0.35	0.28 ± 0.41	−0.072	0.241
100% group	0.15 ± 0.32	0.19 ± 0.38	−0.470	0.394
*t* value	1.213	0.774		
*P* value	0.097	0.324		

Cylinder (D)				
50% group	0.31 ± 0.28	0.29 ± 0.34	0.308	0.145
100% group	0.34 ± 0.41	0.40 ± 0.32	−1.096	0.265
*t* value	−0.339	−2.047		
*P* value	0.412	0.036^*∗*^		

Spherical equivalent (D)				
50% group	0.27 ± 0.31	0.26 ± 0.38	0.052	0.892
100% group	0.25 ± 0.34	0.30 ± 0.33	−0.685	0.787
*t* value	0.674	−0.857		
*P* value	0.251	0.136		

LogMAR				
50% group	0.01 ± 0.03	0.02 ± 0.02	0.042	0.652
100% group	0.01 ± 0.02	0.02 ± 0.03	0.054	0.765
*t* value	0.009	0.012		
*P* value	0.924	0.714		

*Note*. *P-Dist*: distance between the pupil center and the coaxially sighted corneal light reflex. ^*∗*^*P* < 0.05 （the paired *t*-tests were used to detect differences between the 50% group and the 100% group; correlations with different *P-Dist* were determined using the unpaired *t*-test). There were 130 eyes in small-angle *k* group (62 eyes in the 50% group and 68 eyes in the 100% group) and 124 eyes in large-angle k group (65 eyes in the 50% group and 59 eyes in the 100% group).

**Table 3:** Comparison of higher-order aberrations between different *P-Dist* groups at 3 months (the pupil diameter is 5.0 mm).

Category	*P-Dist* < 0.220 mm	*P-Dist* > 0.220 mm	*t* value	*P* value
HOA RMS				
50% group	0.35 ± 0.19	0.29 ± 0.15	0.202	0.514
100% group	0.41 ± 0.23	0.32 ± 0.17	0.944	0.354
*t* value	−1.529	−0.569		
*P* value	0.124	0.253		

Coma				
50% group	0.17 ± 0.12	0.21 ± 0.17	−1.323	0.391
100% group	0.22 ± 0.19	0.34 ± 0.25	−2.017	0.045^*∗*^
*t* value	−1.424	−2.322		
*P* value	0.256	0.021^*∗*^		

Spherical aberration				
50% group	0.09 ± 0.07	0.12 ± 0.09	−1.261	0.354
100% group	0.13 ± 0.12	0.14 ± 0.10	−0.085	0.102
*t* value	−1.525	−0.194		
*P* value	0.247	0.136		

Trefoil				
50% group	0.21 ± 0.12	0.24 ± 0.15	−0.825	0.265
100% group	0.24 ± 0.15	0.30 ± 0.17	−1.434	0.142
*t* value	−0.516	−0.186		
*P* value	0.234	0.258		

*Note*. ^*∗*^*P* < 0.05 (the paired *t*-tests were used to detect differences between the 50% group and the 100% group; correlations with different *P-Dist* were determined using the unpaired *t*-test). There were 130 eyes in small-angle *k* group (62 eyes in the 50% group and 68 eyes in the 100% group) and 124 eyes in the large-angle *k* group (65 eyes in the 50% group and 59 eyes in the 100% group).

## Data Availability

The clinical research data used to support the findings of this study have been deposited in the Dryad repository (doi:10.5061/dryad.hhmgqnkf8). The data used to support the findings of this study are available from the corresponding author upon request.
